# Hibernation Conditions Contribute to the Differential Resistance to Cadmium between Urban and Forest Ant Colonies

**DOI:** 10.3390/ani11041050

**Published:** 2021-04-08

**Authors:** Lauren Jacquier, Mathieu Molet, Céline Bocquet, Claudie Doums

**Affiliations:** 1 Institute of Ecology and Environmental Sciences of Paris (IEES-Paris), UPEC, CNRS, Sorbonne Université, IRD, INRA, 75005 Paris, France; mathieu.molet@sorbonne-universite.fr (M.M.); bocquetceline0@gmail.com (C.B.); 2 Institut de Systématique, Evolution, Biodiversité (ISYEB), Muséum National d’Histoire Naturelle, CNRS, Sorbonne Université, EPHE-PSL, Université des Antilles, 75005 Paris, France; claudie.doums@ephe.sorbonne.fr; 3 Ecole Pratique des Hautes Etudes-Paris Sciences Lettre University, 75014 Paris, France

**Keywords:** common garden, cold, trace metal, urbanization, social insects

## Abstract

**Simple Summary:**

The resistance of organisms to trace metals can have a genetic or a plastic origin. Indeed, differential environmental conditions experienced before the exposure to trace metals could physiologically condition organisms and plastically enhance their subsequent resistance to trace metals. In this study on the ant *Temnothorax nylanderi*, we investigated whether the better cadmium resistance of urban colonies relative to forest colonies could originate from the distinct hibernation conditions that they experienced prior to cadmium exposure. We compared the ability of urban and forest colonies to resist cadmium depending on whether they had hibernated in their respective urban or forest habitats or under a laboratory common garden setup. We found that urban colonies resisted cadmium better than forest colonies when they had hibernated under a common garden. Surprisingly, this difference was not observed between urban and forest colonies that had hibernated in the field, in contrast with a previous study. One reason may be that winter was particularly mild on the year of our experiment. Our results therefore support the idea that urban colonies are genetically adapted to resist trace metals, but that this adaptation is only revealed under specific environmental conditions.

**Abstract:**

Trace metals such as cadmium are found in high concentrations in urban environments. Animal and plant populations living in heavily contaminated environments could adapt to trace metals exposure. A recent study shows that urban populations of the acorn ant *Temnothorax nylanderi* are more resistant to cadmium than their forest counterparts. However, this study was performed using field colonies that had just come out of hibernation. Because urban and forest hibernation environments differ, the differential resistance to trace metals may originate either from differential hibernation conditions or from a different resistance baseline to cadmium. In this study, we tested these two hypotheses using laboratory common garden hibernation conditions. We let urban and forest colonies of the ant *T. nylanderi* hibernate under the same laboratory conditions for four months. After this hibernation period, we also collected field-hibernating colonies and we compared cadmium resistance between urban and forest colonies depending on the hibernation condition. We found a differential response to cadmium under common garden, with urban colonies displaying less larval mortality and lower size reduction of the produced individuals. This suggests a different resistance baseline of urban colonies to cadmium. However, unexpectedly, we did not detect the differential response between urban and forest colonies in the field, suggesting a more complex scenario involving both genetic and environmental influences.

## 1. Introduction

Trace metals at high concentrations have negative impacts on wildlife as they affect cellular and physiological processes (DNA damage, higher oxidative stress, brain inflammation [1,2,3]) and life history traits (body size reduction, decreased birth rate and hatching rate for eggs, higher mortality rate [2,3,4,5,6,7]). High concentrations are mainly associated with human activities such as active or disused mining sites, sewage evacuations into aquatic environments, and in dense cities because of high traffic activity or home heating [8,9,10]. Animal populations living in such contaminated sites can be less sensitive to negative effects of trace metals because of a higher expression of stress-related genes when exposed to trace metals or because of changes in detoxification-linked turnover of proteins [11,12]. For example, in response to metal exposure, some invertebrate populations have higher expression of metallothionein (*Orchesella cincta,* Collembola [12]) heat shock protein (*Chironomus tentans,* Diptera [13]) or antioxidant enzymes, more stable expression of calcium-related stress signaling (*Lumbricus rubellus*, Annelida [14]), and higher secretion or higher turnover of metallothionein protein (*Eisenia fetida*, Annelida [15], and *Hediste diversicolor,* Annelida [11]). These effects have been tested on individuals that had been reared under common garden environments in the laboratory [12,13] or on individuals collected in the field one or two weeks before the start of ecotoxicological assays [11,14,15]. Common garden (i.e., the rearing of individuals from different populations under the same environmental conditions) experiments prove that the higher resistance to trace metals in populations from heavily contaminated soils is a stable response that is not directly induced by the stressful environment and reflects a different baseline resistance that could result from genetic adaptation. However, this method does not allow for the addressing of the question of a modulation of this genetic adaptation by interactions with environmental conditions.

The above-mentioned physiological responses to trace metals are not necessarily stressor-specific and can also be induced by other stressors such as cold and freezing. For instance, a physiological mechanism allowing insects to face winter cold and freezing is “diapause”. Diapause is defined as a “hormonally mediated state of low metabolic activity associated with reduced morphogenesis, increased resistance to environmental extremes and altered or reduced behavioral activity” [16]. Interestingly, some of the anti-stress factors expressed during winter diapause are the same as those expressed in response to trace metal exposure, such as metallothionein, heat shock protein, glutathione, or antioxidant enzymes [16,17,18,19]. Temperature at which diapause occurs impacts the secretion of these anti-stress factors [16,20]. For example, in *Ostrinia nubilalis*, a lepidoptera, larvae entering diapause at cold temperature express high levels of heat shock protein and lower levels of glutathione S transferase compared with larvae that entered diapause at warm temperature, which express higher levels of glutathione S transferase, metallothionein, ferredoxin, and heat shock protein. These differential patterns of gene expression are reinforced with the time spent in the cold environment by diapausing larvae [16]. Temperature can also impact diapause stability, with higher metabolic rate when winter temperatures are warmer or more variable [21]. This negatively impacts winter survival but also long-term survival and fitness of individuals because of lower weight at the end of diapause [21,22]. Thus, winter temperature can have long-term impacts on life history traits and could ultimately affect future resistance to trace metals.

Ants are often used to monitor pollution because they are present in almost all terrestrial ecosystems and because they have long-lived colonies with local foraging, making them prone to local bioaccumulation [23]. Ants are considered as relatively resistant to trace metals pollution, maybe because of their effective metal regulation systems [23]. Most temperate ant species enter diapause during winter, with differences regarding which life stage enters diapause depending on the species [24]. Winter temperature impacts worker survival, with higher or lower worker survival during warm winter, highlighting a sensitivity to hibernation temperature [25,26].

Cadmium is a trace metal with toxic effects on organisms because of its interference with essential metals such as calcium or zinc [27]. For example, organisms exposed to cadmium have decreased hatching rate, longer or stopped development, or reduced body size [4,5,6]. Cadmium is found at high concentrations in cities because of traffic and industrial activities [9,27]. For example, urban wood soils of the city of Paris are in modern times more concentrated in cadmium than adjacent rural wood soils (2.45 mg/kg vs. 0.30 mg/kg, [9]). Genetic adaptation to cadmium pollution has been documented in some arthropod species such as *O. cincta* (collembola) [12], *Drosophila melanogaster* (Diptera) [28], and *Porcellio scaber* (Isopod) [29] by comparing populations living in heavily contaminated sites (ancient mining sites, blast furnace steelworks factories [10]) and rural sites. However there is, to our knowledge, only one study assessing whether urban populations also display some adaptation to this trace metal [30]. Interestingly, a previous study showed that in the ant *Temnothorax nylanderi*, urban colonies are more resistant to cadmium than their forest counterparts, with a weaker negative impact of cadmium on larval survival and body size of emerging workers [30]. However, this study was conducted on colonies that had overwintered in the field. Therefore, the differences observed between urban and forest populations may reflect differences in hibernation environments, as this species nests aboveground and is exposed to variation in climatic environments during winter [31]. For example, in cities, winter air temperature is usually warmer because of the urban heat island effect [32], but at the same time there is less snow cover to protect soil organisms from the cold [33]. Cities also have reduced rain fall and moisture [33]. Because of these different hibernation conditions, gene expression levels of anti-stress factors may differ between city and forest populations, resulting in a differential “preparation” to post-hibernation cadmium exposure.

In this paper, we tested whether the effect of cadmium compared to a control differentially affected the colonies life history traits according to (i) the hibernation conditions (in the field or under common laboratory conditions) and (ii) the origin of the colonies (urban or forest colonies). More specifically, we tested whether the differential colony response to cadmium previously observed between urban and forest populations of the ant *T. nylanderi* [30] is maintained under a common garden (lab) hibernation condition. This would indicate that the differential response is not caused by external environmental factors and that genetic adaptation may have occurred in city populations, even though maternal and epigenetic effects could not be ruled out. Alternatively, the absence of differential response under lab hibernation condition would suggest that the differential response is a plastic response, i.e., there is no genetic differences between urban and forest colonies, and the better cadmium resistance of urban colonies is due to different past environmental conditions (hibernation condition here) that would have enabled them to resist better to cadmium.

## 2. Materials and Methods

The small acorn ant *T. nylanderi* is a common species in Europe, found in both urban and rural habitats. Colonies consist of a few hundred individuals who nest in pieces of dead plant material on the ground (twigs, acorns, chestnuts, etc.), making them easy to collect. Colonies were collected in a park within Paris (Buttes Chaumont, 48°50′59.684″ N 2°21′40.385″ E) for the urban habitat and in a wood 50 km away from Paris (Chantilly, 49°10′59.8″ N 2°28′43.6″ E) for the forest habitat. A previous study [9] found a mean concentration of 2.45 mg/kg of cadmium in Paris urban wood soils, and of approximately 0.30 mg/kg of cadmium in rural wood soils (a proxy of our Chantilly forest soils). We first collected colonies in October 2019 that we used for the common garden (lab) hibernation condition. Just after collection, colonies were brought back to the laboratory and we let them acclimatize for 1 week at 10–15 °C in climatic chamber (reference CTS TP10/600). After the acclimatization period, they were put under artificial overwintering at 4 °C for approximately 4 months (2 November to 9 March). For comparison, the mean temperature from November to early March was 8.1 °C for Paris and 6.9 °C for Chantilly (infoclimat data, available online: https://www.infoclimat.fr (accessed on 4 December 2020)). In early March, lab colonies were warmed up to 10–15 °C for 1 week. At the same time, we collected colonies in the field from the same populations, we brought them back to the laboratory, and we let them acclimatize for 1 week at 10–15 °C (“field colonies”). These colonies had therefore hibernated in the field. The experiment started at the same time for both lab colonies and field colonies after the week at 10–15 °C.

All colonies were reared in 11.5 × 11.5 × 5.5 cm plastic boxes with the lid pierced to let air circulate. Artificial nests were made of 2 microscope slides separated by a 2 mm thin moss chamber, covered by a dark plastic sheet to protect the colonies from the light. Colonies were provided with water ad libitum within a small tube plugged with cotton. We discarded colonies with no queen (41 colonies), with more than 1 queen (17 colonies), or with workers infected by a cestode (4 colonies). Infected workers have a typical pale-yellow coloration [34]. We ultimately kept 72 lab colonies and 70 field colonies.

Two days before starting the experiments, we removed all eggs and larvae from the colonies except for the second instar (S2) larvae, which corresponds to an early developmental stage, and thus all individuals would be exposed to cadmium at the same timing of their development and for the vast majority of their development duration. We counted all workers and remaining S2 larvae. Number of larvae ranged from 2 to 51. The initial number of workers was called “colony size”. Colony size ranged from 9 to 280 workers in lab colonies, and from 11 to 225 in field colonies.

### 2.1. Experimental Design

The experiment started when colonies were put under a 22–27 °C 12–12 h cycle and exposed to 2 different treatments (control and cadmium). Colonies were fed every other day with a mixture made of yoghurt, dried crickets, diluted honey, and vitamins, with 100 µg/g of cadmium in the food (cadmium treatment) or without cadmium (control treatment). This concentration is 50 times higher than the average cadmium concentration in urban wood soils of Paris, but corresponds to the LC_50_ determined in a previous study [30]. Food was provided in excess (approximately 5 g of food per colony). As colonies were fed 3 times a week, this food quantity was enough to last for 2 days whatever the colony size.

We removed newly laid eggs every week. The experiment lasted for 61 days, which corresponds to the maximal duration of larvae development from second-instar to a newly emerged worker at 22–27 °C. Therefore, if some eggs went unnoticed during removal, newly hatched larvae could not have reached adulthood before the end of the experiment. We collected newly emerged workers daily (subsequently called “lab workers”) and stored them in 90° ethanol for further analysis. Those workers are recognizable by their typical pale orange color that they lose after a few days. The collection date was noted, and the total development duration was computed as the number of days between the start of the experiment and the collection date.

Colonies were evenly assigned to control or cadmium treatment so that colony size distribution was the same between the 2 treatments for both lab and field colonies.

### 2.2. Measured Variables

Dead workers were counted every week and their corpses were removed. The worker mortality rate was estimated as the total number of dead workers divided by the number of initial workers in the colony.

As previously observed, some larvae did not grow but stayed alive during the course of the experiment, and were kept within the nest by the workers [30]. These larvae were not extra larvae hatched during the experiment as we removed all newly laid eggs from the nest on a weekly basis. We assumed that these larvae paused their development, as described in other studies, and they were therefore referred to as “paused larvae” [19]. The larval paused development rate was measured as the ratio between the number of paused larvae at the end of the experiment and the initial number of larvae. The number of emergent larvae corresponded to the number of larvae that achieved their development into adults (workers, males, and gynes) at the end of the experiment. The emergence rate was computed as the ratio between the number of emerged adults and the initial number of larvae. The number of dead larvae was obtained by subtracting the number of emerging adults and paused larvae from the initial number of larvae. The larvae mortality rate was computed as the ratio between the number of dead larvae and the initial number of larvae. Therefore, the sum of emergence rate, larval paused development rate, and larvae mortality rate was equal to 1.

We also measured the size of lab workers. We recorded the duration of their development as the number of days between the start of the experiment and their collection time. Heads of the workers were removed and adhered to a double-sided tape. Heads were photographed using a stereomicroscope (Zeiss Discovery.V12). Head width was measured as the eye-to-eye (posterior side) length, a good proxy of the worker size [35], using ImageJ (available online: https://imagej.nih.gov/ij/, accessed on 3 February 2018, [36]). Using a linear mixed effect model, we checked that the development duration was not different depending on hibernation conditions, treatments, or habitats so that differences in head width were not caused by different development durations.

All the above-mentioned measurements are given in Table S1 (for the individual-level variables) and Table S2 (for the colony level variables).

### 2.3. Statistical Analyses

We used R v3.6.2 [37] for all subsequent statistical analysis.

We compared the response to cadmium of city and forest colonies that had overwintered in the field and colonies that had overwintered in common garden in the lab. We tested for the effect of treatment (control or cadmium), habitat (city or forest), and hibernation conditions (field or lab) and their interactions on the 5 response variables (workers and larvae mortality rate, emergence rate, larval paused development rate, lab workers size). We also added colony size, alone or in interaction with treatment, as a covariable. If the cadmium response depends on the habitat but not on hibernation conditions, we expected to find a treatment–habitat interaction with no treatment–habitat–conditions triple interaction.

We used a generalized linear model (GLM) to analyze the effect of the factors on worker mortality rate, larvae mortality rate, larval paused development rate, and emergence rate. We used a quasibinomial logit link as a simple binomial logit link was associated with over dispersion. We built the complete model including all factors and their interactions. We used a backward stepwise procedure to choose the minimum adequate model. The complete model was compared with the same model without the variable of interest. The two models were compared with a log-likelihood test (ANOVA) using χ^2^ or Fisher scores depending on the model. The minimum adequate model was obtained when no more variables could be extracted from the model without significantly changing this model. We obtained the *p*-value for each variable by adding or removing the tested variable from the minimum adequate model and comparing the 2 models using a log-likelihood test.

In contrast with the above-mentioned dependent variables, the head width of lab workers was based on several measurements within each colony. Consequently, we used a mixed model with the colony as a random factor to control for pseudoreplication, but the analyses and statistical tests were the same as above, this time using the nlme package [38].

For all linear models, we visually checked for homoscedasticity and normal distribution of the residuals using plot functions. We used ggplot2 R package [39] for data visualization.

## 3. Results

Thirteen queens died during the course of the experiment (nine lab colonies and four field colonies). We discarded these colonies at the time the queen died, and we removed them from subsequent analyses. Out of the 129 remaining colonies (63 in lab and 66 in field conditions), we collected a total of 999 workers (562 in lab and 437 in field conditions), 327 males (181 in lab and 146 in field conditions), and 87 gynes (63 in lab and 24 in field conditions). Despite this relatively high number of males and gynes, field forest colonies produced only three males and no gyne under cadmium treatment, which prevented us from analyzing data regarding sexuals.

### 3.1. Worker Mortality Rate

**Figure 1:**
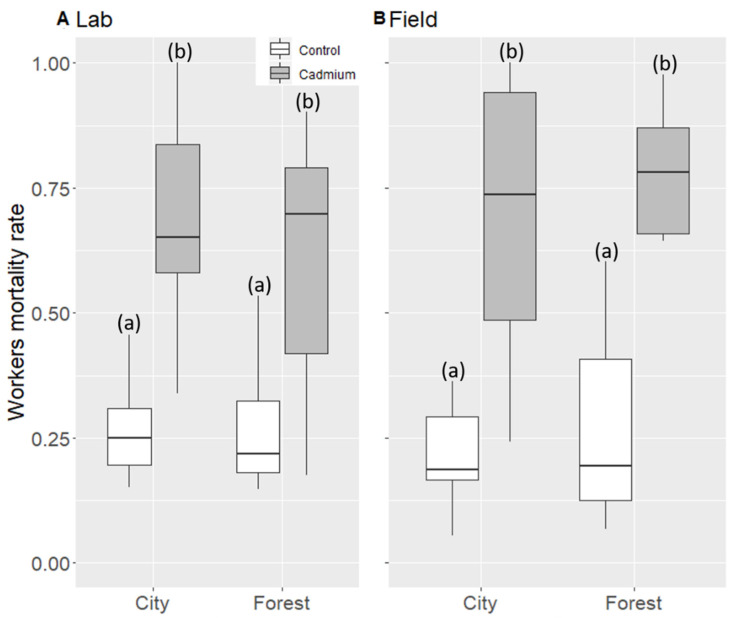
Worker mortality rate in control (white) or cadmium (gray) treatment for lab (**A**) and field hibernation conditions (**B**). For each box, median and quartiles are shown. Different letters represent significant differences (a, b).

Cadmium treatment significantly increased worker mortality rate (+42% in colonies treated with cadmium, Table 1 and Figure 1). This increase in worker mortality rate was similar whatever the habitat (no treatment–habitat interaction, Table 1 and Figure 1) or hibernation conditions (no significant treatment–conditions interaction, Table 1 and Figure 1). The worker mortality rate was not significantly different between the two habitats or hibernation conditions (no conditions or habitat effect, Table 1).

### 3.2. Larvae Mortality Rate

**Figure 2:**
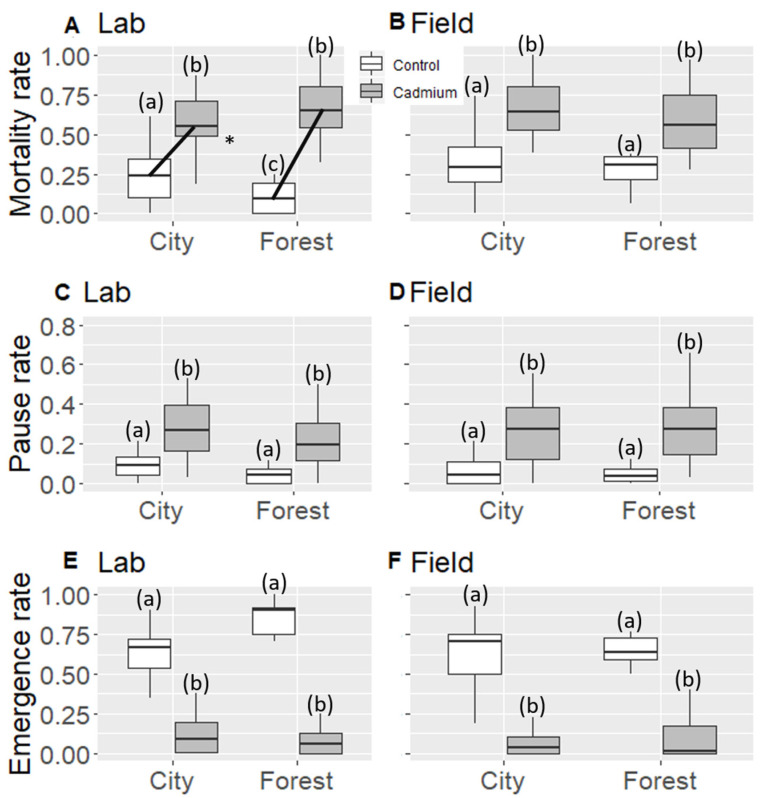
Mortality, larval paused development, and emergence rate of larvae for lab (**A**,**C**,**E**) and field hibernation conditions (**B**,**D**,**F**). White: control treatment, gray: cadmium treatment. For each box, median and quartiles are shown. (*) shows a significant double interaction. Black lines denote the double interaction. Different letters represent significant differences for each lines (a, b).

The larvae mortality rate was higher under cadmium treatment (+36%). The effect of cadmium tended to differ between habitat and hibernation conditions (+32%/+51% mortality for city/forest colonies under lab condition, and +31%/28% for city/forest colonies under field condition), with a triple interaction close to the significant level (treatment–habitat–conditions, F_118,119_ = 3.28, *p* = 0.072). In order to investigate this trend further, we split our dataset according to the hibernation conditions. When considering only the field hibernation condition, cadmium increased larvae mortality rate (+30%, Table 2 and Figure 2), but there was no differential effect of cadmium treatment depending on the habitat, i.e., city colonies did not resist better to cadmium (no treatment–habitat interaction, Table 2 and Figure 2). Interestingly, when considering only the lab hibernation condition, the decrease in larvae mortality rate under cadmium treatment was less pronounced in urban colonies (+32%) than in forest colonies (+51%), i.e., urban colonies were less impacted by cadmium (treatment–habitat interaction, F_56,57_ = 5.33 *p* = 0.024, Table 2 and Figure 2).

### 3.3. Larval Paused Development Rate

The larval paused development rate was higher under cadmium treatment (+20%, Table 3 and Figure 2). Cadmium treatment affected larval paused development rate to a similar extent for the two habitats (no treatment–habitat interaction, Table 3 and Figure 2), i.e., there was no better cadmium resistance in city colonies. Cadmium treatment also affected larval paused development rate to a similar extent for the two hibernation conditions (no significant treatment–conditions interaction, Table 3 and Figure 2). The other tested factors had no significant effects on the larval paused development rate (Table 3).

### 3.4. Emergence Rate

The larvae emergence rate was lower under cadmium treatment (−57%, Table 3 and Figure 2), with a similar extent for the two habitats and two hibernation conditions (no significant triple and double interactions, Table 3 and Figure 2). The larvae emergence rate was higher for colonies hibernating in the lab than in the field (+8%, Table 3).

### 3.5. Size of Lab Workers

Under lab hibernation conditions, we collected 56 city and 32 forest workers in the cadmium treatment, and 203 city and 257 forest workers in the control treatment. Under the field hibernation conditions, we collected 23 city and 36 forest workers in the cadmium treatment, and 207 city and 167 forest workers in the control treatment. The sample size (in terms of number of workers) was therefore unbalanced between cadmium and control treatments.

**Figure 3:**
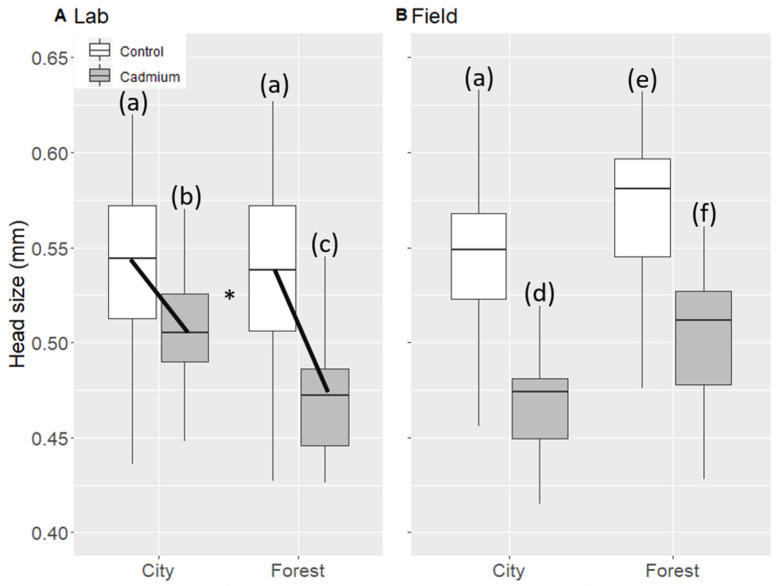
Head size of lab workers in lab (**A**) and field hibernation conditions (**B**). White: control treatment, gray: cadmium treatment. For each box, median and quartiles are shown. (*) shows a significant double interaction. Black lines denote the double interaction. Different letters represent significant differences (a, b, c, d, e, f).

Cadmium treatment decreased the size of lab workers, but the extent of the effect differed depending on the level of both habitat and hibernation conditions (marginally significant treatment–habitat–conditions interaction, X_1_ = 4.17 *p* = 0.041, Figure 3). Because of the marginally significant triple interaction, we split our dataset according to the hibernation conditions. When considering only the field hibernation condition, the decrease in size of lab workers under cadmium treatment was similar in both types of habitat, i.e., city colonies did not respond better to cadmium than the forest colonies (no significant treatment–habitat interaction, Table 2 and Figure 3). However, when considering only the lab hibernation condition, the decrease in size of lab workers under cadmium treatment was less pronounced in city than in forest habitat (significant treatment–habitat interaction, X_1_ = 4.06 *p* = 0.043, Table 2 and Figure 3). Moreover, forest colonies produced significantly larger workers than city colonies (Table 2 and Figure 3).

## 4. Discussion

Populations living in trace-metal contaminated environments such as mining site or cities are often more tolerant to trace metals than populations living in pollution-free environments [12,30,40]. In the ant *T. nylanderi*, Jacquier et al. [28] showed that urban populations are less sensitive to cadmium than their forest counterparts, with a lower larvae mortality rate, a lower decrease in emergence rate, and a lower size reduction of lab workers when exposed to cadmium. We proposed two alternative hypotheses regarding the enhanced cadmium resistance of city colonies: (i) if it is due to a baseline resistance of urban populations, it should still occur following common garden (lab) hibernation; (ii) if it comes from a plastic response caused by hibernation in an urban environment, it should disappear following common garden (lab) hibernation. We found that the lower cadmium sensitivity of urban colonies was maintained after four months of hibernation under laboratory common garden, suggesting a different baseline level of cadmium resistance that could reflect genetic adaptation. As in Jacquier et al. [28], urban colonies reared under laboratory common garden for several months had a lower larvae mortality rate and a lower size reduction of lab workers relative to forest colonies. However, in contrast with Jacquier et al., we did not find a higher emergence rate in laboratory common garden urban colonies under cadmium. For worker mortality rate and larval paused development rate, the treatment with cadmium had a similar negative impact for the city and forest colonies. Unexpectedly, there was no differential cadmium resistance between city and forest colonies that hibernated in the field, in contrast with previous results in the same localities [30]. Overall, our results suggest that the differential response to cadmium between city and forest colonies is not directly caused by the different hibernation conditions in the field since it was still observed under lab hibernation conditions. However, the unexpected fact that we were unable to detect a differential response in the field in this study suggests that it could be expressed only under specific hibernation conditions as discussed below.

In a previous study [30], the differential response to cadmium between urban and forest colonies was found for one pair of locations in 2017 (Paris), and for three other pairs of locations in 2018 (Paris, Lyon, and Bordeaux). Relative to forest colonies, urban colonies displayed a lower worker size reduction, a higher emergence rate [30], and a lower larvae mortality rate in response to cadmium (only 2018 data available, personal data). The pattern was the same in all four comparisons, suggesting that this differential response was stable. In agreement with this, in our study, we found the same pattern under laboratory hibernation condition but quite surprisingly we did not detect any differential response in the field. The sample size in Jacquier et al. was larger (148 field colonies compared with 66 field colonies in our study), and thus we may have lacked statistical power in the present study. However, graphical tendencies seem to point a lack of effect or a reversed effect of cadmium (with lower larvae mortality rate and lower worker size reduction in forest colonies in response to cadmium), and therefore it is unlikely that the different pattern found in this study originated from a lack of statistical power. Another hypothesis is that the hibernation environment plays a role in the expression of the differential response to cadmium even though it does not directly explain the differential baseline response of city and forest colonies. More specifically, we suggest that cold winter is necessary to induce the differential response observed between forest and city colonies. Insect survival during overwintering is often related to temperature. For example, warmer winter increases mortality rate in insects such as the rose-gallin wasp *Dilopepsis spinosa* [41] or the boreal ant *Formica aquilonia* [26], probably because of higher metabolic rate that depletes fat reserves. On the other hand, some species seem to perform better under warm temperature, such as the ant *Lasius niger*, for which workers have lower mortality rate at warm temperatures [25]. In our study, mean winter temperature in 2019/2020 was 8.1 °C in Paris city and 6.9 °C in Chantilly forest (infoclimat data, https://www.infoclimat.fr/stations-meteo/analyses-mensuelles.php (accessed on 4 December 2020)), higher than in 2017/2018 (city 6.2 °C, forest 4.7 °C) and 2018/2019 (city 6.3 °C, forest 5.2 °C) and higher than in the laboratory common garden environment (4 °C for both habitats). Cold winters could favor the strongest individuals, as suggested by Haatenen et al. [25]. Therefore, toxicological assays may have been performed on colonies in poor condition. This, however, was not well-supported by our mortality data, with a similar mortality rate for colonies whatever the hibernation temperature (cold in the lab and warm in the field). In addition, we removed parasitized colonies from our experiment, which were more susceptible to be in poor conditions. Further studies performed on both unparasitized and parasitized colonies could be interesting to investigate ecological interactions between parasites and trace metal resistance. Cold stress triggers secretion of anti-stress proteins that are also involved in trace metal detoxification or resistance. For instance, in the earthworms *Dendrobanea octaedra*, freezing of body fluids triggers the secretion of metallothionein, a well-known protein group involved in trace metal detoxification [42]. In *Ostrinia nubilalis*, the gene expression patterns of cold-acclimated diapausing individuals during diapause initiation, maintenance, and termination differ from those of warm-acclimated diapausing individuals, with higher quantity and stability of mRNA in cold-acclimated individuals at the end of the diapause [16]. Proteins coded by those mRNA are involved in temperature-related stress (heat shock protein, HSP) but interestingly also in antioxidative defenses and metal detoxification (metallothionein, thioredoxin, ferredoxin), which may help individuals to better cope with future trace-metal related stress. We therefore propose that the field hibernation conditions in 2019/2020 were not cold enough to trigger the expression of differential response to cadmium. The absence of differential cadmium response between city and forest for field colonies may therefore have been due to warmer temperature during winter diapause. In contrast, our laboratory hibernating conditions were colder than what was observed in the field (4 °C, i.e., 1–2 °C colder than in winter 2017/2018 and 3–4 °C colder than in winter 2019/2020). This could explain why the differential response was found in the common garden experiment and not in the field in our study. However, in contrast with Jacquier et al. (2020), this study was only conducted on a single urban/forest pair of habitats. Therefore, we cannot conclude whether the importance of hibernation conditions is a general pattern or is specific to the Paris location. However, a genetic study highlighted the very high genetic homogeneity of *T. nylanderi* populations throughout France. In addition, the differential effect of cadmium between city and forest habitats was found to be similar in three distant locations in France (Jacquier et al. 2020). These two studies support the idea that our results could be generalized to most large cities in France, although further studies with replicated locations would be needed to confirm this.

Interestingly, even though the hibernation temperature in the laboratory common garden was even cooler than the average field temperature, it seemed to be not so stressful, at least for post-hibernation performances, as the worker mortality rate and the size of lab-workers were similar in Jacquier et al. [28] and in our study, whatever the hibernating conditions. This indicates that the threshold under which cold temperature has negative effects on post-hibernation traits had not been reached under our common garden hibernation experiment. This is not surprising given that the temperature of the common garden (4 °C) is the same order of magnitude as the mean winter temperature commonly observed under such latitude (available online: https://www.infoclimat.fr/climato/, accessed on 4 December 2020).

The differential response to cadmium found between forest and city colonies under common hibernating laboratory environments supports the idea that this differential response could be at least in part genetically determined. A genetic adaptation to trace metal has already been shown in other insect populations living in ancient mining sites [12], in fish populations living in polluted water [43], and in urban bird populations [40]. A recent genetic analysis found 19 discriminating single-nucleotide polymorphisms (SNPs) between urban and forest populations of *T. nylanderi* [44]. One of these SNPs may be involved in the differential response between urban and forest colonies to cadmium exposure, but this however remains highly speculative. More studies are needed to disentangle the effects of genetic adaptation from maternal or epigenetics effects. In any case, our results suggest that urban populations have higher basal levels of anti-stress gene expression than their forest counterparts, in line with the higher expression of immune response, antioxidative, detoxification, and repair systems genes in urban populations of other species (*Parus major* birds [45], *Anopheles* spp. mosquitoes [46], *Peromyscus leucopus* rodents [47]). Interestingly, those anti-stress genes are involved in trace metal resistance. For instance, detoxification proteins such as metallothionein or cytochrom P450 (CYP450) improve trace metal resistance by binding metals [27,48]. As trace metals also increase oxidative stress, enhancing the expression of antioxidant defenses (e.g., glutathione S transferase or thioredoxin) or DNA repair systems (e.g., EXO1 endonuclease [2]) may help to cope with such pollutants. As those classes of genes are overexpressed in urban populations for quite divergent species, we can hypothesize that urban populations of *T. nylanderi* also overexpressed such types of genes. This hypothesis would merit further attention and could be tested by quantifying the differential expression of stress-related genes, known to be sensitive to temperature, between urban and forest populations.

## 5. Conclusions

In conclusion, this study highlights the interconnection between genetic and environmental components in the trace metal response of urban and forest ant colonies for one specific area (Paris). We hypothesized that the better resistance to cadmium of urban colonies is only found under a specific environmental condition, i.e., cold winter. In the light of future climate change and warmer winter temperatures, it is of particular importance to assess whether adaptation to a specific anthropogenic disturbance is stable or sensitive to other environmental disturbances, possibly impeding adaptation processes.

## Figures and Tables

**Table 1:** Summary of the statistical analyses on workers testing the effects of hibernation conditions (lab and field), type of habitat (city and forest), and treatment (control or cadmium) on the workers mortality rate. T:H:C means treatment–habitat–conditions triple interaction. Bold number indicates significant *p*-value.

	Worker Mortality Rate
T:H:C	F_125__,119_ = 0.87
	*p* = 0.52
Treatment–Habitat	F_125,123_ = 0.0099
	*p* = 0.90
Treatment–Conditions	F_125,123_ = 1.98
	*p* = 0.14
Habitat–Conditions	F_125,122_ = 0.75
	*p* = 0.52
Treatment	**F_125,126_ = 4.91**
	***p* = 0.028**
Habitat	F_125,124_ = 0.058
	*P* = 0.81
Conditions	F_125,124_ = 1.45
	*p* = 0.23
Colony Size–Treatment	**F_125,126_ = 6.2**
	***p* = 0.014**
Colony Size	NA

**Table 2:** Summary of statistical analyses performed by splitting dataset according to hibernation conditions on larvae mortality rate and size of lab workers. For each model, we tested the effect of habitat (city and forest), treatment (control or cadmium), and colony size. Left side of the column: laboratory hibernation condition (Lab), right side of the column: field hibernation condition. Bold number indicates significant *p*-value.

	Larvae Mortality Rate		Size of Lab Workers	
Treatment–Habitat	**Lab**	Field	Lab	Field
	**F_56,57_ = 5.33**	F_62,60_ = 0.18	X_1_ = 4.06	X_1_ = 0.50
	***p* = 0.024**	*p* = 0.83	*p* = 0.043	*p* = 0.47
Treatment	Z = −7.59	F_62,63_ = 18.41	t_65_ = 6.52	X_1_ = 50.73
	*p* < 0.001	*p* < 0.0001	*p* < 0.001	*p* < 0.0001
Habitat	Z = −1.30	F_61,62_ = 0.20	t_63_ = 0.88	X_1_ = 12.84
	*p* = 0.19	*p* = 0.65	*p* = 0.38	*p* = 0.0003
Colony Size–Treatment	F_57,58_ = 1.69	F_62,63_ = 4.57	X_1_ = 0.17	X_2_ = 3.05
	*p* = 0.19	*p* = 0.036	*p* = 0.67	*p* = 0.22
Colony Size	F_58,59_ = 3.63	NA	X_1_ = 4.46	X_1_ = 2.91
	*p* = 0.061		*p* = 0.035	*p* = 0.088

**Table 3:** Summary of statistical analyses on larvae testing the effects of hibernation conditions (lab and field), type of habitat (city and forest), and treatment (control or cadmium) for larval pause development rate and emergence rate. T:H:C means treatment–habitat–conditions triple interaction. Bold number indicates significant *p*-value.

	Pause Rate	Emergence Rate
T:H:C	F_121,127_ = 0.78	F_126,121_ = 1.29
	*p* = 0.58	*p* = 0.26
Treatment–Habitat	F_127,125_ = 1.64	F_126,124_ = 1.33
	*p* = 0.19	*p* = 0.26
Treatment–Conditions	F_127,125_ = 0.37	F_126,125_ = 0.040
	*p*=0.69	*p* = 0.84
Habitat–Conditions	F_127,124_ = 0.87	F_126,124_ = 1.09
	*p* = 0.46	*p* = 0.33
Treatment	**F_127,128_ = 100.14**	**F_126,127_ = 257.29**
	***p* < 0.0001**	***p* < 0.0001**
Habitat	F_127,126_ = 1.69	F_126,125_ = 1.82
	*p* = 0.19	*p* = 0.17
Conditions	F_127,126_ = 0.034	**F_126,127_ = 6.64**
	*p* = 0.85	***p* = 0.011**
Colony Size–Treatment	F_127,125_ = 0.17	F_126,124_ = 0.50
	*p* = 0.84	*p* = 0.60
Colony Size	F_127,126_ = 0.32	F_126,125_ = 0.23
	*p* = 0.57	*p* = 0.63

## Data Availability

Data are available in the Supplementary Materials section (Tables S1 and S2).

## Supplementary Materials

The following are available online at https://www.mdpi.com/article/10.3390/ani11041050/s1: Table S1: Emergent head width (individual-level data). Table S2: Summary of the colony-level data (worker mortality, larval mortality, larval paused, emergence).
